# Meetings of Societies

**Published:** 1898-03

**Authors:** 


### MEETINGS OF SOCIETIES.

### Bristol Medico-Chirurgical Society.

*December 8th*, 1897.

Dr. J. E. SHAW, President, in the Chair.

Allusion was made to the ability displayed by the Medical Officers of Health in detecting the cause of the recent outbreak of enteric fever in Clifton. (See December number, p. 382.)

Dr. J. MICHELL CLARKE read a paper on "The Treatment of Enteric Fever." (For this and the discussion which took place upon it, see pp. 15-30.)

Mr. T. CARWARDINE showed a specimen of calcareous empyema. The man, aged 50, was admitted under the care of Dr. Shaw at the Royal Infirmary with symptoms of pleuritic effusion. Under nitrous oxide an opening was made in the 8th intercostal space, and twenty-three ounces of non-fœtid pus came away. The patient did not improve, and on resecting a portion of the 10th rib under chloroform some three weeks later much calcareous material could be felt with the finger. He refused any further operation, and died from hemorrhage six weeks after admission. *Post-mortem* the lung was collapsed, the pleura thickened and coated on its whole internal surface by a thick crust of calcareous deposit, including the upper surface of the diaphragm. The sour milk-like odour of the discharge towards the last suggested that lactic acid fermentation was taking place within the cavity.

*January 12th*, 1898,

Dr. J. E. SHAW, President, in the Chair.

Dr. F. H. EDGEWORTH showed (1) a case of Hodgkin's disease. The patient was 17 years of age, and had been ill only seven weeks. The lymph glands in the neck, axillæ and groins were enlarged, as were also the spleen, which extended 2 in. below the level of the umbilicus, and the liver. The interesting feature was the occurrence of a bilirubin jaundice, not hitherto described in this complaint. This was probably due to blood destruction from capillary hemorrhages. This view was supported by the fact that whilst the percentage of corpuscles was 38, that of hæmoglobin was 45; (2) A case of wrist-drop due to lead. The exceptional features were that the biceps and triceps jerks were exaggerated, and the musculo-spiral nerves were tender in both arms. This was probably due to the fact that the neuritis was in an early stage, and had not been described previously in cases of neuritis due to lead.

Mr. W. H. HARSANT showed a specimen of papillomatous disease of the bladder. The patient had been operated on four years previously, when the growth was removed by supra-pubic cystotomy. He remained free from the disease for three years, but it eventually recurred, and caused death by suppurative pyelitis.

Dr. BARCLAY BARON remarked on the removal of papillomatous growths from the larynx, and recommended the use of guarded laryngeal forceps with cutting edges, if forceps are employed for the removal of these growths. He also showed a large polypus removed from the naso-pharynx, which had its attachment within the nostril and projected into the mouth, and was snared off through the mouth. He recommended making traction on the growth previous to passing the snare wire up behind the palate by means of a blunt-ended pair of forceps, rather than of a sharp vulsellum, as these growths are apt to be cystic, and the vulsellum tears out the wall. No interference with the soft palate is usually necessary.

Dr. T. FISHER showed two specimens of extensive ulceration of the colon, a small spleen weighing only two ounces, two specimens of septic pneumonia, a case of thrombosis of one division of the renal vein, and a small abscess in the liver. These were all associated with enteric fever.—Dr. JAMES SWAIN referred to a case of abscess of the liver complicating typhoid fever in a child nearly 5 years of age. On December 8th—when suffering from a relapse, and when in the sixth week of the disease—the child had a rigor, for which no cause could be assigned. During the month the patient had seven rigors, and a slight eversion of the left costal cartilages was noticed. On January 2nd there was a distinct swelling in the epigastrium, obviously connected with the left lobe of the liver. The following day an incision was made in the parietes, an exploring needle passed into the liver to confirm the diagnosis of abscess, the liver stitched to the surface, the abscess (which appeared as large as a cricket ball) opened, and a drainage tube placed in the cavity. A culture of the fluid showed numerous colonies of actively motile bacilli of typhoid with some staphylococci. Since operation the child had made a steady improvement, the temperature being below normal.

The discussion on Dr. Michell Clarke's paper on "The Treatment of Enteric Fever" was continued (see p. 30).

Dr. J. G. SWAYNE read a paper on "Occipital Presentations." (This will be printed in a future number.)

*February 9th*, 1898.

Dr J. E. Shaw, President, in the Chair.

Dr. Barclay Baron showed a case of epithelioma of the larynx in a man aged 68 years. There was thickening of the arytenoid cartilage, the ary-epiglottic fold, the ventricular band on the right side, and the fossa outside the arytenoid is filled up with a nodular growth. The symptoms complained of are some difficulty of swallowing, difficulty of breathing, and extreme hoarseness, all of which have existed for six months and are increasing. The question of removal of half the larynx was considered, Mr. Morton seeing the patient in consultation; but inasmuch as the disease is extrinsic in this case, both laterally and on the œsophageal side having extended outside the limits of the larynx, and inasmuch as it is impossible to say to what extent the œsophagus is involved, it was thought unwise to undertake it. Tracheotomy may be urgently necessary, as there are paroxysms of severe dyspnœa at night, and not only does this procedure shield the patient from this danger, but it actually retards the progress of the growth by giving physiological rest to the larynx, which, owing to the glottis being encroached upon by the growth, is submitted to much wear and tear in sucking in the air under such difficulty.

The President showed a lad, aged 14, the subject of cerebellar ataxia. This malady, which Marie first described, is probably only a sub-group of cases originally included in Friedreich's ataxia. From the latter it is distinguished by the presence of increased knee-jerks, dyschromatopsia, and occasional eye-ground changes, and by the absence of scoliosis, the two forms possessing in common the quasi-alcoholic gait and speech. This case was remarkable for the rapidity of development and the youthfulness of the subject.

Mr. W. M. Barclay showed two patients on whom he had performed extensive resections of tarsal bones, and read a paper on "Tubercular Disease of the Foot." In the one case, after an unsuccessful attempt to cure tubercular disease starting in the scaphoid by gouging its interior, he, on 3rd September, 1896, excised the remains of the scaphoid and the whole astragalus; completely scooped out the os calcis, cuboid, external and middle cuneiforms—leaving only the shells of these; removed the articular cartilage and cancellous tissue of the external malleolus, cutting away pulpy synovial membrane. The wound healed entirely, and the patient had now a shapely foot, on which he was beginning to bear his weight. The second case came up in an advanced stage of disease, with discharging sinuses, a dorsal abscess, and necrosis of the terminal phalanx of the great toe. The scaphoid, the cuneiforms, the cuboid, and the entire fifth metatarsal bone were excised, and the bases of the remaining metatarsals were gouged freely. The astragalus and os calcis appeared to be healthy. The tip of the great toe was amputated. This operation was done on March 22nd, 1896; and when the patient was last seen shortly before Christmas, 1897, the wound remained closed, and the foot seemed well. Now, however, it was found that disease had reappeared in the astragalus: but in this situation it would doubtless be amenable to further treatment. In view of these results, and remembering the connections of the scaphoid, through its synovial sac, with the three cuneiforms, the second and third metatarsals, and the cuboid, and directly with the head of the astragalus behind, he would discard all scraping operations in early scaphoid disease, and advocate its clean excision. If, however, on undertaking this operation it were found that the disease had spread beyond it, he advised excision of all the bones connected with it through the synovial membrane (as enumerated above), with excision of the head of the astragalus if the posterior articular surface of the scaphoid had been invaded. When the disease was more advanced, scraping and gouging operations might still give good results if *thoroughly* done: the affected tarsal bones should be excised, except the astragalus and os calcis, in which gouging was very successful. The possibility of getting rid of the synovial disease by scraping depended on the manner of its implication: if it had become infected by rupture of a tubercular focus into its cavity, the softened surface, to which for some time the tubercular deposits were limited, could be scraped away; but if the infection had spread from the edge of the bone, or if it were primary in the synovial membrane, the whole thickness of that membrane was the seat of tubercles, and it was too tough to be scraped. In cases of this kind, where the scaphoid group was infected, Mr. Watson Cheyne's suggestion of a clean excision *en bloc* by section through the heads of the astragalus, os calcis, and the bases of the metatarsals would probably give the best result. Where scraping operations were adopted, the raw bony surfaces and the wound cavity should be swabbed with pure liquefied carbolic acid, large quantities of iodoform should be introduced, and the whole cavity firmly packed with strips of iodoform gauze. Sepsis materially increased the risk of local reinfection and of general dissemination. Pure carbolic acid was the strongest means we had of combating this, and iodoform, in addition to its antiseptic properties, had a distinct influence over tubercular deposits. He urged the importance of continuing the strict antiseptic treatment of these cases until all sinuses had *completely* healed: that, after the first firm plugging of the cavities, the subsequent packings should only lightly fill the diminishing space, and be finally left out when only a sinus remained, so as not to keep it from closing: that rest, while invaluable here as in tubercular disease of other parts, should not be too long persevered in, to the exclusion of operation, unless unmistakable evidence of improvement were observed; but it should be *absolute*, for which purpose some form of stiff bandage, such as plaster-of-Paris, enclosing the foot, ankle, and leg, was the only efficient apparatus; and, if possible, this should be combined with the horizontal position. Advancing disease did not always give signs of its presence, when such absolute rest was maintained, so that it was necessary to inspect the foot at short intervals.—Mr. C. A. Morton referred to those fortunate cases of tuberculous disease of the tarsus, in which the removal of one or two sequestra of tuberculous bone quickly brought the disease to an end; and also to a case of disease in the joint between the os calcis and astragalus, which was treated by erasion of the joint after separation of the two bones, when the tendo Achillis had been freely divided and the incision carried outwards along the upper border of the os calcis as for excision of that bone. Mr. Morton also referred to the difficulty in diagnosis of the seat of the tubercular disease, when situated in the lower end of the tibia and sinuses open around the astragalus.—In answer to Mr. Morton's remarks, Mr. Barclay said he had not attempted to give a complete account of tuberculosis of the foot. It was well known that the astragalus and os calcis were the most favourable sites for such disease in the foot, and thorough scraping, gouging, and other partial operations generally yielded excellent results in their case.

Professor Fawcett showed: (1) Three original plaster models of the bony ear, one of the middle ear and its surroundings, one of the labyrinth, external aspect, and a third of the interior of the vestibule, cochlea and the semicircular canals; (2) Three dissections of the brain, mounted by a new method in plaster-of-Paris, and described the method of mounting. One specimen showed especially the fascia dentata from below. The other was a preparation of the whole length of the fornix, it showed also the bundle of Vicq d'Azyr. The third was a preparation showing the velum interpositum, veins of Galen, and their formation. The veins and choroid plexuses were coloured. These preparations were all preserved in a two per cent. solution of formol, which had been found best for the purpose as it did not penetrate the cement; (3) A series of humeri with epiphyses from the first year up to 21; and three mounted specimens of the skeleton of the hand, two of which were of the age of from 10 to 11 years and showed the various epiphyses. These specimens of epiphyses were illustrative of a complete series of the whole body which have been prepared in the anatomical department during the past five months, and constitute a very valuable if not unique collection.

Dr. J. Lacy Firth showed a dermoid removed from a woman, aged 38, together with the ovaries and Fallopian tubes and several omental dermoids of varying size. He read notes of the case and remarked upon the universal adhesions which had made the tumour difficult to remove. He remarked that the tumour appeared to be oöphoronic in origin. The omental dermoids were secondarily peritoneal, and had arisen primarily in ovarian tissue. They had been separated by adhesions. The patient was discharged in a healthy state in five weeks, but it was very likely that other peritoneal dermoids had been left behind.—Dr. Aust Lawrence advised the early removal of these tumours on account of the dangers from adhesions to bladder and pressure on ureter leading to surgical kidney.—Mr. J. Ewens detailed a case of dermoid cyst involving the left ovary, which was shown some years ago at this Society, removed from a girl, aged 7 years, in the Children's Hospital. It was about the size of a large orange, and contained bones of upper and lower jaw with six teeth, and a portion of scalp to which was attached a coil of hair measuring thirty inches when fully extended, a most unusual length as considered by Mr. Bland Sutton, who showed it at the Obstetrical Society of London. Patient made a rapid recovery, and when seen several years after she was free from any abdomimal affection.—Mr. C. A. Morton suggested that perhaps the cyst was not oöphoronic in origin, as the ovary did not seem expanded by the growth of a large cyst, but might have been merely adherent to the cyst.—Mr. T. Carwardine remarked on the rarity and interest of the specimen, and thought the main cyst must have originated near, but not in the ovary itself, say in one of the tubules of the broad ligament. The origin of ovarian dermoids is difficult of explanation embryologically, as the genital ridge arises in the mesoblastic cœlom; but its continuity with epiblast is remotely established through the primitive streak of the embryo. The multiplicity of the dermoids might be accounted for by dissociated "rests" of the genital ridge persisting in the peritoneal cavity and there developing.—Dr. Firth, replying to Mr. Morton and Mr. Carwardine, said that in spite of their criticism he was still inclined to regard the tumour as of oöphoronic origin. He thought that the cyst in its early stages had probably been very prominent on the surface, and that the early growth had been towards the surface, thus exerting a minimum effect npon the ovarian stroma.

Dr. F. H. Edgeworth read a paper on "Hysterical Paroxysmal Œdema." (This will be printed in a future number.)

J. Paul Bush, *Hon. Sec.*

### Plymouth Medical Society.

*December 13th*, 1897.

Dr. C. Aldridge, President, in the Chair.

Mr. J. N. Morris showed a young, well-developed and muscular man with a condition of skin over the pectorals which he regarded as morphœa. Other members regarded it as simply over-stretching of the skin from excessive muscular exertion whilst rowing, with rapid muscular development, analogous to the lineæ albicantes after pregnancy.

Mr. J. R. Rolston showed a case of keratoglobus, and pointed out the seriousness of the outlook, these cases usually developing glaucoma.

Mr. Aldous gave an interesting demonstration of the urethroscope on the living subject, and handed round coloured plates representing the conditions most usually revealed by its aid.

Mr. Ryan showed a case which had recently developed signs and symptoms of a cerebral tumour; these, however, under the influence of iodides had almost disappeared at the time of exhibition.

Mr. Woollcombe showed, for Mr. Swain, a popliteal aneurysm removed from the limb of an old man, aged 78, after amputation through the thigh. The femoral had been ligatured at the apex of Scarpa's triangle twelve years before by the late Sir Wm. Savory.

Dr. F. Bushnell read a note on Torre's method of preserving pathological specimens.

Dr. Pethybridge showed a specimen and a microscopic slide of the radula of a limpet. Similar bodies had been passed *per rectum* in large numbers by a patient, and caused considerable intestinal irritation.

---

*January 29th*, 1898.

Dr. C. Aldridge, President, in the Chair.

Mr. J. S. Ward showed a fine, healthy-looking girl whose left arm terminated abruptly two inches below the elbow, there being no trace of fingers or nails on the stump; the upper epiphyses of the radius and ulna were present, and apparently a portion of the diaphyses. The patient's mother attributed the deformity to an impression received during fifth month of pregnancy. Mr. Lucy, Mr. Whiteford, and Mr. Woollcombe regarded it as a case of intra-uterine amputation, probably by the umbilical cord.

Mr. Lucy showed a man, aged 26, who about three and a half years previously had received a cut, severing some of the flexor tendons an inch above the wrist; the tendons were sutured, but ever since he had been troubled with corns over the terminal phalanges of the index and second fingers, occasionally breaking down into excavated ulcers. He had anæsthesia corresponding to the distribution of the median nerve; the nails of the affected fingers were becoming incurved, and Mr. Lucy thought there were indications that the terminal phalanges were becoming absorbed.

Dr. F. BUSHNELL read notes of a case in which death had occurred in a young adult from fæcal impaction.

Mr. J. E. SQUARE showed a concretion removed from the umbilicus which was about ¾ inch long and ¼ inch diameter, and consisted of free fatty acids and a substance somewhat resembling cholesterine, which was probably koprosterine. It was regarded as an intestinal concretion, which probably had ulcerated out of a Meckel's diverticulum.

Mr. ALDOUS showed a partially perforate hymen from a primipara.

Mr. WHITEFORD showed a supernumerary tooth from a case of cleft-palate.

Mr. HAMILTON showed three bean-shaped bodies, removed *post mortem* from the bladder of a man who died suddenly after a compound fracture of the leg. A further report was promised after a microscopical examination.

Mr. WOOLLCOMBE (for Mr. Swain) showed a kidney removed from a single woman with a large growth springing from the central pyramid, probably carcinoma.

W. L. WOOLLCOMBE, *Hon. Sec.*

